# Two psammobiont species of *Anomalohalacarus* (Acari, Halacaridae) from South Korea

**DOI:** 10.3897/BDJ.12.e135719

**Published:** 2024-10-07

**Authors:** Jong Hak Shin, Cheon Young Chang, Jimin lee

**Affiliations:** 1 Ocean Climate Response & Ecosystem Research Department, Korea Institute of Ocean Science & Technology, Busan, Republic of Korea Ocean Climate Response & Ecosystem Research Department, Korea Institute of Ocean Science & Technology Busan Republic of Korea; 2 Department of Biomedical Science, Daegu University, Gyeongsan, Republic of Korea Department of Biomedical Science, Daegu University Gyeongsan Republic of Korea

**Keywords:** *Anomalohalacarusangustus* sp. nov., *Anomalohalacarusbiformis*, arenicolus, marine mites, meiofauna, taxonomy

## Abstract

**Background:**

The genus *Anomalohalacarus* Newell, 1949, which is known to occur exclusively inhabiting sand, has not been previously reported from Korea. During a recent survey of the meiofauna from several intertidal sandy beaches in South Korea, we found both sexes of two psammobiont halacarids, *Anomalohalacarusangustus* sp. nov. and *A.biformis* Abé, 1996.

**New information:**

Both species are similar to each other in sharing an undivided posterior dorsal plate, three and two setae on the anterior and posterior epimeral plates, respectively, three bipectinate setae on tibia IV, and four ventral setae on tarsus I. However, *A.angustus* sp. nov. is distinguished from *A.biformis* by the absence of areolae posteriorly on the anterior dorsal plate, a much more slender posterior dorsal plate, a pair of subgenital setae on the genital foramen in females, and seven branched perigenital setae on the genitoanal plate in males. The feature of branched perigenital setae in males is reported for the first time in the genus *Anomalohalacarus*. The Korean specimens of *A.biformis* agree well with the original description from Japan, except for the number of ventral bipectinate setae on tibia II. This study presents (re)descriptions of both species with detailed illustrations and provides a dichotomous key to *Anomalohalacarus* species, including the new species, based on morphological characters. The genus *Anomalohalacarus* is reported for the first time in South Korea.

## Introduction

Psammobiont environments play a crucial role in the ecological framework of coastal ecosystems (Schwinghamer 1981, Reise 2001). These habitats are characterised by unique physical and chemical properties, including high permeability, variable particle size and fluctuating salinity and oxygen levels (Jansson 1967). They offer protection from predators, a rich supply of detritus and microorganisms for food and a stable environment for reproduction and development (Fenchel 1978). The organisms inhabiting these environments have evolved a range of adaptations to thrive in these microhabitats, including small bodies, elongated shapes, and specialised locomotory and sensory structures that allow efficient navigation of interstitial spaces (Higgins and Thiel 1988).

Among marine halacarid mites, several genera almost exclusively inhabit sandy habitats: *Acarochelopodia* Angelier, 1954, *Actacarus* Schulz, 1937, *Anomalohalacarus* Newell, 1949, *Arenihalacarus* Abé, 1991, and *Scaptognathides* Monniot F., 1972 (Abé 1991, Bartsch 1993, Bartsch 2004). They also possess soft, slender, concave bodies enhancing their adherence to sand grains, reduced body plates, slender legs, and reduced or absent pigments in corneas (Bartsch 1991, Bartsch 1993, Giere 1993).

In Korea, 22 marine halacarid species have been documented from various habitats since 2003. Among these, only five appear to be genuine psammobionts: *Actacarusornatus* Shin, Chang and Lee, 2024, *A.pacificus* Bartsch, 1979 (Shin et al. 2024), *Copidognathuscerberoideus* Bartsch, 1991 (Chatterjee et al. 2004), *Scaptognathusteuriensis* Abé, 1990 and *S.magnus* Abé, 1990 from subtidal sediment (Lee and Chang 2017, Lee et al. 2020).

In this study, we describe or redescribe two psammobiont halacarid species, *Anomalohalacarusangustus* sp. nov. and *A.biformis* Abé, 1996, found in the intertidal sandy beaches of the coast in South Korea. We also provide detailed illustrations and descriptions of both species, along with keys to known species in the genus *Anomalohalacarus*, including the present new species. This is the first record of the genus *Anomalohalacarus* from Korea.

## Materials and methods

**Figure 1.**
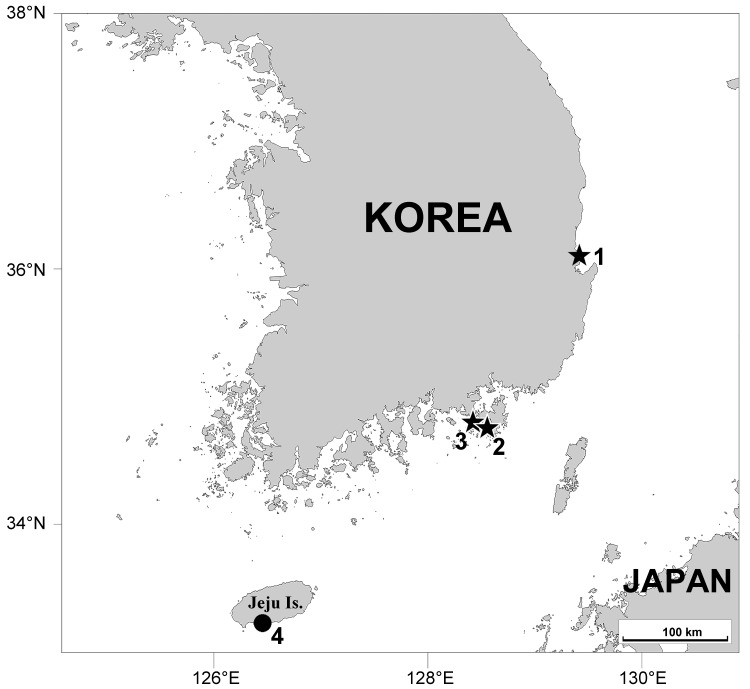
A map showing localities. **1** Chilpo Beach, Pohang; **2** Gujora Beach, Geoje; **3** Sulyug Beach, Tongyeong; **4** Jungmunsaekdal Beach, Jeju-do. Symbols as follows: ★ *Anomalohalacarusangustus* sp. nov., ● *A.biformis*.

Sediments were collected using a suction device after digging in several sand puddles about 0.5–1 m deep with a shovel on intertidal beaches at four localities on the coast of South Korea (Fig. 1). Samples gathered in a bucket were anaesthetised using 7.5% magnesium chloride or tap water for 30–60 min, filtered through a 64 μm-diameter mesh, and then immediately fixed in 80% ethanol or 5% formalin. Detailed methods for preparing slide specimens and drawings are described in our previous study (Lee et al. 2023). Scale bars in the figure are given in micrometres (μm).

The type specimens are deposited at the National Marine Biodiversity Institute of Korea (MABIK), Seocheon, Korea. Some specimens are kept at the Marine Interstitial fauna Resources Bank (MInRB) of the Korea Institute of Ocean Science & Technology (KIOST), Busan, Korea.

Terminology and abbreviations in the text and figure captions follow Bartsch (2006): AD = anterior dorsal plate; AE = anterior epimeral plate; ds-1–ds-6 = the first-to-sixth dorsal setae on idiosoma; GA = genito-anal plate; glp-1–glp-4 = first-to-fourth gland pores on idiosoma; GO = genital opening; GP = genital plate(s); OC = ocular plate(s); P-1–P-4 = first-to-fourth palpal segments; pas = parambulacral setae; PD = posterior dorsal plate; PE = posterior epimeral plate; pgs = perigenital setae; sgs = subgenital setae.

## Taxon treatments

### Anomalohalacarus angustus

Shin, Chang and Lee sp. nov.

B55A2BC1-0604-5F72-9BA1-651B14BCAE75

60D22EBF-06DC-41A8-B892-AF7D9E187883

*Anomalohalacarus* Newell, 1949
Type species: *Anomalohalacarusanomalus* (Trouessart, 1894)

#### Materials

- **Type status:** Holotype. **Occurrence:** individualCount: 1; sex: female; lifeStage: adult; preparations: dissected, mounted in glycerine on H-S slide; occurrenceID: 4A860B8C-956E-53AC-A1A6-5C8E89EF3528; **Taxon:** scientificName: Anomalohalacarusangustus; kingdom: Animalia; phylum: Arthropoda; class: Arachnida; order: Trombidiformes; family: Halacaridae; genus: Anomalohalacarus; specificEpithet: angustus; scientificNameAuthorship: Shin, Chang & Lee; **Location:** higherGeography: Northwest Pacific Ocean; continent: East Asia; country: Korea (the Republic of); countryCode: KR; stateProvince: Gyeongsangbuk-do; municipality: Pohang-city; locality: Chilpo Beach, Heunghee-eup; verbatimDepth: 0.5–1 m; verbatimCoordinates: 36°07'59"N 129°23'57"E; **Identification:** identifiedBy: Shin, Chang & Lee; dateIdentified: 2024; **Event:** samplingProtocol: suction; eventDate: 26/04/2023; **Record Level:** institutionID: MABIK CR00257793; institutionCode: Marine Biodiversity Institute of Korea; basisOfRecord: PreservedSpecimen
- **Type status:** Paratype. **Occurrence:** individualCount: 1; sex: male; lifeStage: adult; preparations: mounted in glycerine on H-S slide; occurrenceID: 879F85D4-A825-544B-8000-A6668FE095A2; **Taxon:** scientificName: *Anomalohalacarusangustus*; kingdom: Animalia; phylum: Arthropoda; class: Arachnida; order: Trombidiformes; family: Halacaridae; genus: Anomalohalacarus; specificEpithet: *angustus*; scientificNameAuthorship: Shin, Chang & Lee; **Location:** higherGeography: Northwest Pacific Ocean; continent: East Asia; country: Korea (the Republic of); countryCode: KR; stateProvince: Gyeongsangbuk-do; municipality: Pohang-city; locality: Chilpo Beach, Heunghee-eup; verbatimDepth: 0.5–1 m; verbatimCoordinates: 36°07'59"N 129°23'57"E; **Identification:** identifiedBy: Shin, Chang & Lee; dateIdentified: 2024; **Event:** samplingProtocol: suction; eventDate: 26/04/2023; **Record Level:** institutionID: MABIK CR00257794; institutionCode: Marine Biodiversity Institute of Korea; basisOfRecord: PreservedSpecimen
- **Type status:** Paratype. **Occurrence:** individualCount: 1; sex: female; lifeStage: adult; preparations: mounted in glycerine on H-S slide; occurrenceID: 3F900439-5D5F-5893-8C7B-D12B1F8406D3; **Taxon:** scientificName: *Anomalohalacarusangustus*; kingdom: Animalia; phylum: Arthropoda; class: Arachnida; order: Trombidiformes; family: Halacaridae; genus: Anomalohalacarus; specificEpithet: *angustus*; scientificNameAuthorship: Shin, Chang & Lee; **Location:** higherGeography: Northwest Pacific Ocean; continent: East Asia; country: Korea (the Republic of); countryCode: KR; stateProvince: Gyeongsangbuk-do; municipality: Pohang-city; locality: Chilpo Beach, Heunghee-eup; verbatimDepth: 0.5–1 m; verbatimCoordinates: 36°07'59"N 129°23'57"E; **Identification:** identifiedBy: Shin, Chang & Lee; dateIdentified: 2024; **Event:** samplingProtocol: suction; eventDate: 26/04/2023; **Record Level:** institutionID: MABIK CR00257795; institutionCode: Marine Biodiversity Institute of Korea; basisOfRecord: PreservedSpecimen
- **Type status:** Paratype. **Occurrence:** individualCount: 1; sex: male; lifeStage: adult; preparations: mounted in glycerine on H-S slide; occurrenceID: 5963AC3E-403D-5602-BB64-E42B60E57386; **Taxon:** scientificName: *Anomalohalacarusangustus*; kingdom: Animalia; phylum: Arthropoda; class: Arachnida; order: Trombidiformes; family: Halacaridae; genus: Anomalohalacarus; specificEpithet: *angustus*; scientificNameAuthorship: Shin, Chang & Lee; **Location:** higherGeography: Northwest Pacific Ocean; continent: East Asia; country: Korea (the Republic of); countryCode: KR; stateProvince: Gyeongsangbuk-do; municipality: Pohang-city; locality: Chilpo Beach, Heunghee-eup; verbatimDepth: 0.5–1 m; verbatimCoordinates: 36°07'59"N 129°23'57"E; **Identification:** identifiedBy: Shin, Chang & Lee; dateIdentified: 2024; **Event:** samplingProtocol: suction; eventDate: 26/04/2023; **Record Level:** institutionID: MABIK CR00257796; institutionCode: Marine Biodiversity Institute of Korea; basisOfRecord: PreservedSpecimen
- **Type status:** Paratype. **Occurrence:** individualCount: 1; sex: male; lifeStage: adult; preparations: mounted in glycerine on H-S slide; occurrenceID: 71CEE945-3FB9-559C-9F54-26F7E1A34437; **Taxon:** scientificName: *Anomalohalacarusangustus*; kingdom: Animalia; phylum: Arthropoda; class: Arachnida; order: Trombidiformes; family: Halacaridae; genus: Anomalohalacarus; specificEpithet: *angustus*; scientificNameAuthorship: Shin, Chang & Lee; **Location:** higherGeography: Northwest Pacific Ocean; continent: East Asia; country: Korea (the Republic of); countryCode: KR; stateProvince: Gyeongsangbuk-do; municipality: Pohang-city; locality: Chilpo Beach, Heunghee-eup; verbatimDepth: 0.5–1 m; verbatimCoordinates: 36°07'59"N 129°23'57"E; **Identification:** identifiedBy: Shin, Chang & Lee; dateIdentified: 2024; **Event:** samplingProtocol: suction; eventDate: 26/04/2023; **Record Level:** institutionID: MABIK CR00257797; institutionCode: Marine Biodiversity Institute of Korea; basisOfRecord: PreservedSpecimen
- **Type status:** Paratype. **Occurrence:** individualCount: 1; sex: female; lifeStage: adult; preparations: mounted in glycerine on H-S slide; occurrenceID: 9E5317B9-7BD6-5F6D-9F29-9554DEC19FEA; **Taxon:** scientificName: *Anomalohalacarusangustus*; kingdom: Animalia; phylum: Arthropoda; class: Arachnida; order: Trombidiformes; family: Halacaridae; genus: Anomalohalacarus; specificEpithet: *angustus*; scientificNameAuthorship: Shin, Chang & Lee; **Location:** higherGeography: Northwest Pacific Ocean; continent: East Asia; country: Korea (the Republic of); countryCode: KR; stateProvince: Gyeongsangbuk-do; municipality: Pohang-city; locality: Chilpo Beach, Heunghee-eup; verbatimDepth: 0.5–1 m; verbatimCoordinates: 36°07'59"N 129°23'57"E; **Identification:** identifiedBy: Shin, Chang & Lee; dateIdentified: 2024; **Event:** samplingProtocol: suction; eventDate: 26/04/2023; **Record Level:** institutionID: MInRB-Hl20-S006; institutionCode: Marine Interstitial fauna Resources Bank; basisOfRecord: PreservedSpecimen
- **Type status:** Paratype. **Occurrence:** individualCount: 1; sex: male; lifeStage: adult; preparations: mounted in glycerine on H-S slide; occurrenceID: 94E1D3FE-765A-50A2-84C1-43F186EEDA48; **Taxon:** scientificName: *Anomalohalacarusangustus*; kingdom: Animalia; phylum: Arthropoda; class: Arachnida; order: Trombidiformes; family: Halacaridae; genus: Anomalohalacarus; specificEpithet: *angustus*; scientificNameAuthorship: Shin, Chang & Lee; **Location:** higherGeography: Northwest Pacific Ocean; continent: East Asia; country: Korea (the Republic of); countryCode: KR; stateProvince: Gyeongsangbuk-do; municipality: Pohang-city; locality: Chilpo Beach, Heunghee-eup; verbatimDepth: 0.5–1 m; verbatimCoordinates: 36°07'59"N 129°23'57"E; **Identification:** identifiedBy: Shin, Chang & Lee; dateIdentified: 2024; **Event:** samplingProtocol: suction; eventDate: 26/04/2023; **Record Level:** institutionID: MInRB-Hl20-S007; institutionCode: Marine Interstitial fauna Resources Bank; basisOfRecord: PreservedSpecimen
- **Type status:** Paratype. **Occurrence:** individualCount: 1; sex: male; lifeStage: adult; preparations: mounted in glycerine on H-S slide; occurrenceID: 95FAE6B4-DA3D-563D-964C-8E0E8789E8A2; **Taxon:** scientificName: *Anomalohalacarusangustus*; kingdom: Animalia; phylum: Arthropoda; class: Arachnida; order: Trombidiformes; family: Halacaridae; genus: Anomalohalacarus; specificEpithet: *angustus*; scientificNameAuthorship: Shin, Chang & Lee; **Location:** higherGeography: Northwest Pacific Ocean; continent: East Asia; country: Korea (the Republic of); countryCode: KR; stateProvince: Gyeongsangbuk-do; municipality: Pohang-city; locality: Chilpo Beach, Heunghee-eup; verbatimDepth: 0.5–1 m; verbatimCoordinates: 36°07'59"N 129°23'57"E; **Identification:** identifiedBy: Shin, Chang & Lee; dateIdentified: 2024; **Event:** samplingProtocol: suction; eventDate: 26/04/2023; **Record Level:** institutionID: MInRB-Hl20-S008; institutionCode: Marine Interstitial fauna Resources Bank; basisOfRecord: PreservedSpecimen
- **Type status:** Paratype. **Occurrence:** individualCount: 1; sex: male; lifeStage: adult; preparations: mounted in glycerine on H-S slide; occurrenceID: 599E0D65-6CAA-5B02-8549-B44A4AB5CB01; **Taxon:** scientificName: *Anomalohalacarusangustus*; kingdom: Animalia; phylum: Arthropoda; class: Arachnida; order: Trombidiformes; family: Halacaridae; genus: Anomalohalacarus; specificEpithet: *angustus*; scientificNameAuthorship: Shin, Chang & Lee; **Location:** higherGeography: Northwest Pacific Ocean; continent: East Asia; country: Korea (the Republic of); countryCode: KR; stateProvince: Gyeongsangbuk-do; municipality: Pohang-city; locality: Chilpo Beach, Heunghee-eup; verbatimDepth: 0.5–1 m; verbatimCoordinates: 36°07'59"N 129°23'57"E; **Identification:** identifiedBy: Shin, Chang & Lee; dateIdentified: 2024; **Event:** samplingProtocol: suction; eventDate: 26/04/2023; **Record Level:** institutionID: MInRB-Hl20-S009; institutionCode: Marine Interstitial fauna Resources Bank; basisOfRecord: PreservedSpecimen
- **Type status:** Other material. **Occurrence:** individualCount: 1; sex: female; lifeStage: adult; preparations: mounted in glycerine on H-S slide; occurrenceID: 85DBD274-A2A1-50D1-9A62-8E8B40555A94; **Taxon:** scientificName: *Anomalohalacarusangustus*; kingdom: Animalia; phylum: Arthropoda; class: Arachnida; order: Trombidiformes; family: Halacaridae; genus: Anomalohalacarus; specificEpithet: *angustus*; scientificNameAuthorship: Shin, Chang & Lee; **Location:** higherGeography: Northwest Pacific Ocean; continent: East Asia; country: Korea (the Republic of); countryCode: KR; stateProvince: Gyeongsangnam-do; municipality: Geoje-city; locality: Gujora Beach, Gujora-ri, Irun-myeon; verbatimDepth: 0.5–1 m; verbatimCoordinates: 34°48'36"N 128°41'11"E; **Identification:** identifiedBy: Shin, Chang & Lee; dateIdentified: 2024; **Event:** samplingProtocol: suction; eventDate: 13/07/2022; **Record Level:** basisOfRecord: PreservedSpecimen
- **Type status:** Other material. **Occurrence:** individualCount: 1; sex: male; lifeStage: adult; preparations: mounted in glycerine on H-S slide; occurrenceID: 9DA22569-2898-59BD-A49B-63A0318CADAD; **Taxon:** scientificName: *Anomalohalacarusangustus*; kingdom: Animalia; phylum: Arthropoda; class: Arachnida; order: Trombidiformes; family: Halacaridae; genus: Anomalohalacarus; specificEpithet: *angustus*; scientificNameAuthorship: Shin, Chang & Lee; **Location:** higherGeography: Northwest Pacific Ocean; continent: East Asia; country: Korea (the Republic of); countryCode: KR; stateProvince: Gyeongsangnam-do; municipality: Geoje-city; locality: Gujora Beach, Gujora-ri, Irun-myeon; verbatimDepth: 0.5–1 m; verbatimCoordinates: 34°48'36"N 128°41'11"E; **Identification:** identifiedBy: Shin, Chang & Lee; dateIdentified: 2024; **Event:** samplingProtocol: suction; eventDate: 13/07/2022; **Record Level:** basisOfRecord: PreservedSpecimen
- **Type status:** Other material. **Occurrence:** individualCount: 1; sex: male; lifeStage: adult; preparations: mounted in glycerine on H-S slide; occurrenceID: A32A825B-D46F-56A7-A5C3-E88285DD0DF5; **Taxon:** scientificName: *Anomalohalacarusangustus*; kingdom: Animalia; phylum: Arthropoda; class: Arachnida; order: Trombidiformes; family: Halacaridae; genus: Anomalohalacarus; specificEpithet: *angustus*; scientificNameAuthorship: Shin, Chang & Lee; **Location:** higherGeography: Northwest Pacific Ocean; continent: East Asia; country: Korea (the Republic of); countryCode: KR; stateProvince: Gyeongsangnam-do; municipality: Geoje-city; locality: Gujora Beach, Gujora-ri, Irun-myeon; verbatimDepth: 0.5–1 m; verbatimCoordinates: 34°48'36"N 128°41'11"E; **Identification:** identifiedBy: Shin, Chang & Lee; dateIdentified: 2024; **Event:** samplingProtocol: suction; eventDate: 13/07/2022; **Record Level:** basisOfRecord: PreservedSpecimen
- **Type status:** Other material. **Occurrence:** individualCount: 1; sex: male; lifeStage: adult; preparations: mounted in glycerine on H-S slide; occurrenceID: EAE612B1-C053-538F-8B26-FD6DEA94B223; **Taxon:** scientificName: *Anomalohalacarusangustus*; kingdom: Animalia; phylum: Arthropoda; class: Arachnida; order: Trombidiformes; family: Halacaridae; genus: Anomalohalacarus; specificEpithet: *angustus*; scientificNameAuthorship: Shin, Chang & Lee; **Location:** higherGeography: Northwest Pacific Ocean; continent: East Asia; country: Korea (the Republic of); countryCode: KR; stateProvince: Gyeongsangnam-do; municipality: Tongyeong-city; locality: Sulyug Beach, Sanyang-eup; verbatimDepth: 0.5 m; verbatimCoordinates: 34°49'09"N 128°26'22"E; **Identification:** identifiedBy: Shin, Chang & Lee; dateIdentified: 2024; **Event:** samplingProtocol: suction; eventDate: 09/05/2024; **Record Level:** basisOfRecord: PreservedSpecimen
- **Type status:** Other material. **Occurrence:** individualCount: 1; sex: male; lifeStage: adult; preparations: mounted in glycerine on H-S slide; occurrenceID: 2543AEC1-3283-5591-8D2B-705927077E24; **Taxon:** scientificName: *Anomalohalacarusangustus*; kingdom: Animalia; phylum: Arthropoda; class: Arachnida; order: Trombidiformes; family: Halacaridae; genus: Anomalohalacarus; specificEpithet: *angustus*; scientificNameAuthorship: Shin, Chang & Lee; **Location:** higherGeography: Northwest Pacific Ocean; continent: East Asia; country: Korea (the Republic of); countryCode: KR; stateProvince: Gyeongsangnam-do; municipality: Tongyeong-city; locality: Sulyug Beach, Sanyang-eup; verbatimDepth: 0.5 m; verbatimCoordinates: 34°49'09"N 128°26'22"E; **Identification:** identifiedBy: Shin, Chang & Lee; dateIdentified: 2024; **Event:** samplingProtocol: suction; eventDate: 09/05/2024; **Record Level:** basisOfRecord: PreservedSpecimen

#### Description

**Female** (holotype)

**Figure 2.**
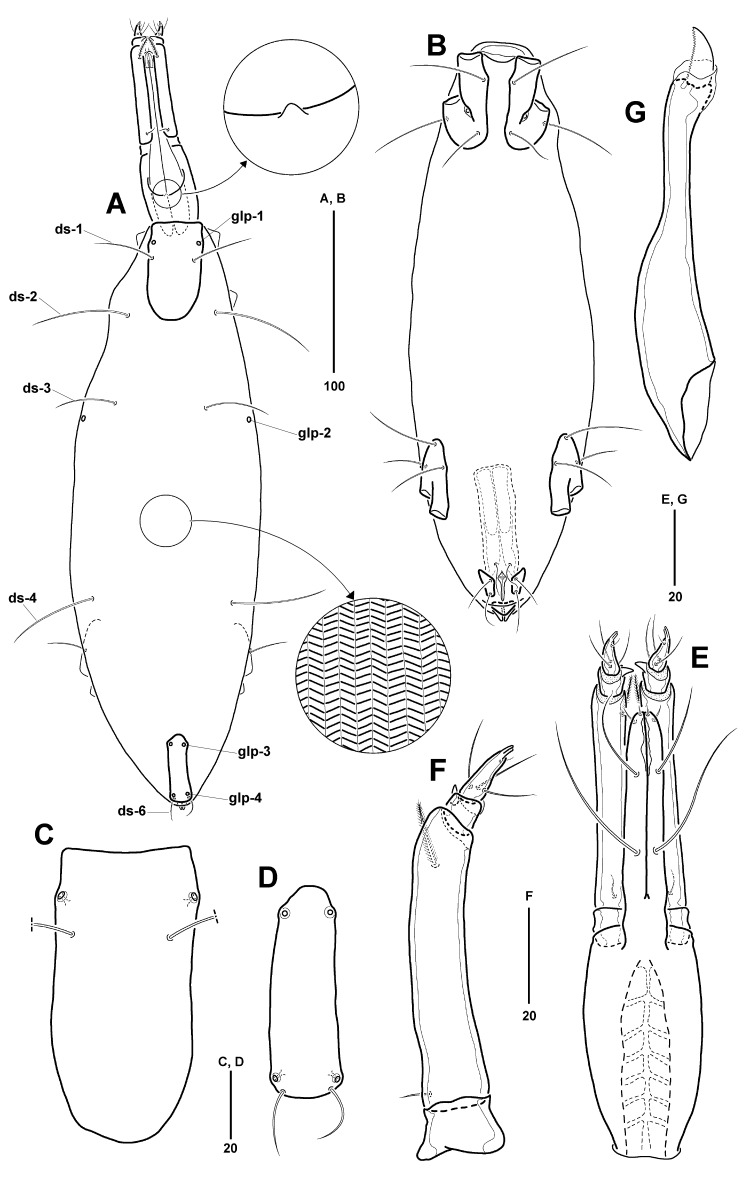
*Anomalohalcarusangustus* sp. nov., female (MABIK CR00257793). **A** Idiosoma, dorsal; **B** Idiosoma, ventral; **C** AD, dorsal; **D** PD, dorsal; **E** Gnathosoma, ventral; **F** Palp, lateral; **G** Chelicerae, lateral.

Idiosoma (Fig. 2A) elongate, 364 µm long (360–372 µm, mean = 365 µm, n = 4), 103 µm wide (103–107 µm, mean = 105 µm, n = 4), length-to-width ratio about 3.52 (3.43–3.52, mean = 3.48, n = 4). Dorsal plates (AD and PD) with smooth surface, each plate clearly separated by membranous cuticle; OC absent. Membranous cuticle between AD and PD 71% of idiosoma, covered with parallel wavy striae transversely; ornamented with 1 pair of glp (glp-2) and three pairs of ds (ds-2–ds-4); glp-2 located at anterior third of idiosoma dorsolaterally.

AD (Fig. 2A, C) 84 µm long (82–87 µm, mean = 84 µm, n = 4), about 0.23 times as long as idiosoma, 42 µm wide (40–44 µm, mean = 42 µm, n = 4), length-to-width ratio 1.98; rectangular-shaped with truncated anterior margin and slightly convex posterior margin; with 1 pair of glp-1 and ds-1; glp-1 located at anterior 15% of AD laterally.

PD (Fig. 2A and D) undivided, smaller than AD, 0.74 times as long as AD, 0.52 times as wide as AD; 62 µm long (60–68 µm, mean = 63 µm, n = 4), about 0.17 times as long as idiosoma, 22 µm wide (22–26 µm, mean = 24 µm, n = 4), length-to-width ratio 2.79; elongate rectangular-shaped with convex anterior and blunt posterior margins; with a pair of ds-6 and 2 pairs of glp (glp-3 and glp-4), each glp placed at anterior 14% and 91% of PD, respectively.

Five pairs of dorsal setae (Fig. 2A): ds-1 located at anterior 29% of AD; 3 pairs of ds (ds-2–ds-4) situated on membranous cuticle dorsolaterally, at anterior 16%, 31%, and 65% of idiosoma, respectively; ds-2 at level of posterior margin of AD; ds-2 and ds-4 slightly longer than others, similar to length of AD; ds-5 absent; ds-6 at posterior margin of PD.

All ventral plates (Fig. 2B) small and separated by membranous cuticle with striae similar to those on dorsal surface of idiosoma, except between left and right plates of AE covered with smooth membranous cuticle. AE (Fig. 2B and Fig. 4A) 58 µm long (58–64 µm, mean = 60 µm, n = 4), about 0.16 times as long as idiosoma, 26 µm wide (26–29 µm, mean = 27 µm, n = 4), length-to-width ratio 2.20; divided into left and right plates by membranous cuticle without striae; distance between plates widest at 21 μm near insertion of leg I, narrowest at 12 μm near posterior ventral seta; each plate bearing 2 ventral, 1 lateral setae, and 1 epimeral pore; posterior ventral seta (0.5 times as long as AE) shorter than others (0.8 times as long as AE); epimeral pore inserted at anterior 61% of AE.

PE (Fig. 2B) 49 µm long (46–53 µm, mean = 50 µm, n = 4), about 0.13 times as long as idiosoma, 18 µm wide (17–20 µm, mean = 18 µm, n = 4), length-to-width ratio 2.76; located at anterior 68–81% of idiosoma; divided into two plates, with 1 pair of dorsolateral setae and 2 pairs of ventral setae; foremost ventral seta located at anterior corner of PE and another seta at anterior 37% of PE, respectively.

GP (Fig. 2B and Fig. 4B) divided into 3 plates, consisting of 1 pair of anterior genital plates and 1 posterior genital plate; anterior genital plates sub-rectangular, situated lateral to genital foramen, 17 µm long (16–19 µm, mean = 17 µm, n = 4), 9 µm wide (8–10 µm, mean = 9 µm, n = 4), length-to-width ratio of 1.96; posterior genital plate inverted triangular, between genital and anal foramina, 7 µm long (6–9 µm, mean = 7 µm, n = 4), 16 µm wide (13–20 µm, mean = 17 µm, n = 4), length-to-width ratio of 0.46; 3 pairs of pgs in genital area; foremost pgs situated on membranous cuticle anterior to genital foramen; 2 pairs of pgs on anterior genital plates. Genital foramen (Fig. 4B) 19 µm long (17–21 µm, mean = 19 µm, n = 4), 4 µm wide (4–5 µm, mean = 4 µm, n = 4); slender, matchstick-shaped, pointed at anterior tip, widest at anterior 14%; 1 pair of sgs at widest genital foramen; genital groove placed between posterior genital plate and anal sclerites. Ovipositor (Fig. 2B) tube-shaped, its anterior tip not reaching anterior margin of PE.

Gnathosoma (Fig. 2A and E) slender, 117 µm long (114–124 µm, mean = 118 µm, n = 4), about 0.32 times as long as idiosoma, 31 µm wide (30–33 µm, mean = 31 µm, n = 4), length-to-width ratio of 3.73; without areolae and costae on surface. Rostrum (Fig. 2E) 62 µm long, as long as gnathosomal base, its tip not reaching to distal end of P-2; 4 pairs of rostral setae, consisting of 2 pairs of short rostral setae (proto- and deutorostral setae) on distal tip of rostrum and 2 pairs of long rostral setae (trito- and basirostral setae) located at anterior 26% and 59% of ventral surface of rostrum, respectively. Palp (Fig. 2F) with 4 segments, 11, 63, 8 and 16 µm, respectively; P-1 trapezoid, without setae and spines; P-2 longest, with 1 short proximal and 1 plumose distal setae dorsally, and distal seta slightly longer than proximal one; P-3 shortest, with 1 cuticle spine dorsomedially; P-4 conical, with 3 proximal and 1 tiny distal setae, and terminal tip bifurcated. Chelicera (Fig. 2G) long, 113 µm long, consisting of basal segment and movable digit; basal segment, 99 µm long, with slender distal part (36%) and broadened proximal part; movable digit, 16 µm long, with about 20 fine denticles along dorsal surface. Tectum (Fig. 2A) very small, slightly protruding at middle of anterior margin.

**Figure 3.**
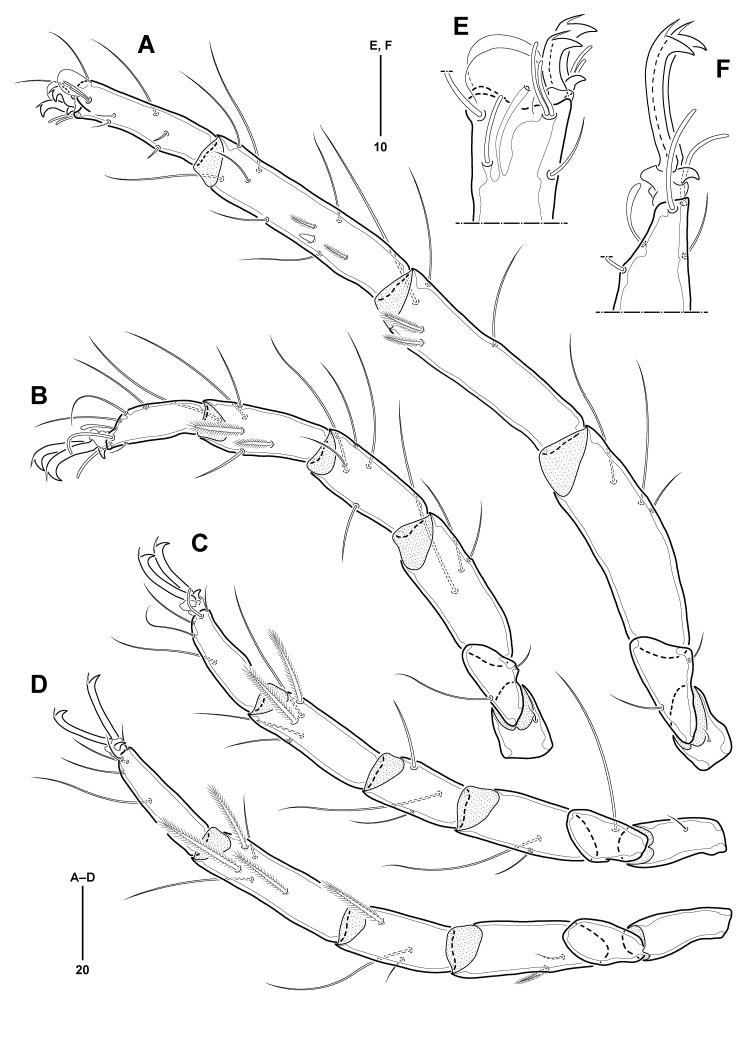
*Anomalohalcarusangustus* sp. nov., female (MABIK CR00257793). **A–D** Legs I–IV, ventromedial; **E** Tip of tarsus I, lateral; **F** Tip of tarsus II, lateral.

All legs (Fig. 3A–D) slender, legs I–IV 270, 168, 169, and 190 µm long, respectively; leg I longest and legs II–IV 0.62, 0.63, and 0.70 times as long as leg I, respectively. Chaetotaxy of legs as follows: trochanters 1-1-1-0; basifemora 2-2-1-0; telofemora 3-3-2-2; genua 5-5-3-3; tibiae 10-6-5-5; tarsi (excluding pas and famulus) 8-5-3-3; number of bipectinate setae on telofemora to tibiae I–IV (none in other segments): telofemora 0-0-0-1; genua 2-0-0-1; tibiae 2-2-2-3. Trochanters shortest in legs I–II. Basifemora shortest in legs III–IV; basifemur III with 1 long lateral seta, more than twice as long as corresponding segment. All genua shorter than preceding segment (telofemur), except for genu I similar in length to telofemur I. All tibiae longer than preceding segment (genu), except for tibiae I and II as long as preceding segment; tibia I with 2 bipectinate setae and 1 small spine ventromedially. All tarsi slightly shorter than preceding segment (tibia); tarsus I (Fig. 3E) with 1 pair of fossary lamellae protruded distally; with 1 solenidion, 1 famulus, 3 dorsal, 4 ventral setae, and 1 pair of doublet eupathid pas; tarsus II (Fig. 3F) with 1 slender claviform solenidion, 3 dorsal and 1 ventral setae, and 1 pair of singlet eupathid pas; tarsus III (Fig. 3C) with 3 dorsal setae and 1 pair of pas consisting of 1 medial eupathid and 1 lateral filiform pas; tarsus IV (Fig. 3D) with 3 dorsal setae and 1 pair of filiform pas; all tarsi with a pair of lateral claws bearing 1 dorsal accessory process and with single median claw; lateral claws of tarsus I shorter than others.

**Male**

**Figure 4.**
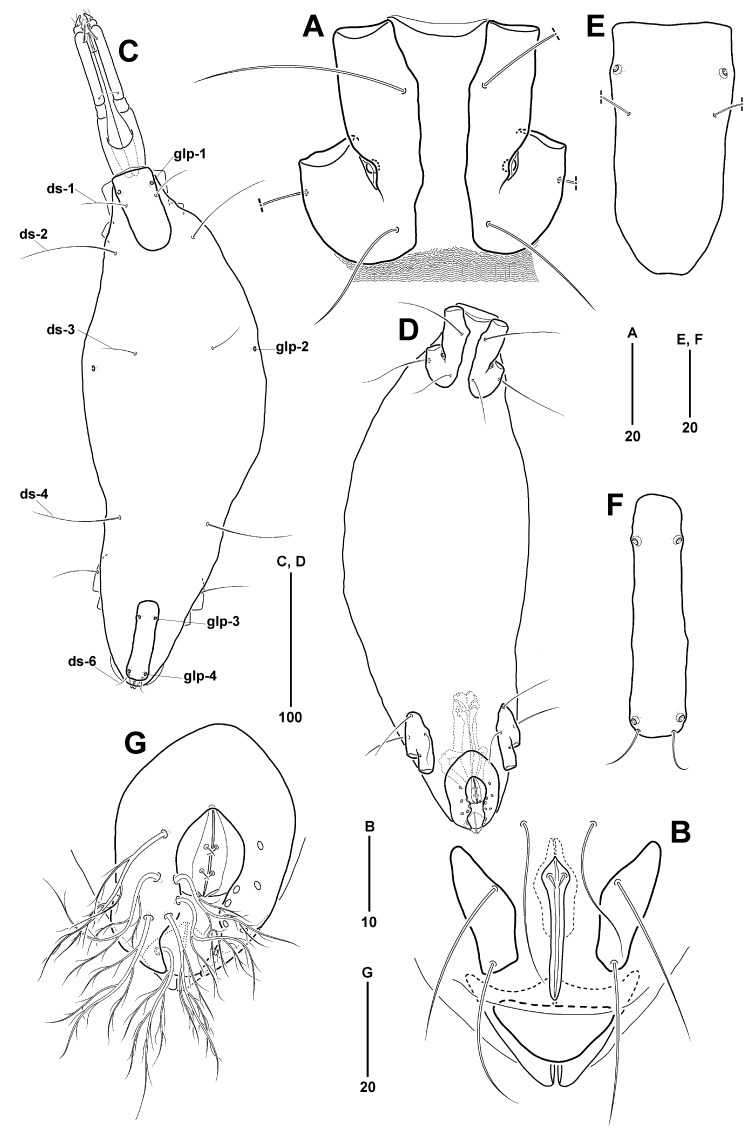
*Anomalohalcarusangustus* sp. nov. A, B, Female (MABIK CR00257793). **A** AE, ventral; **B** GP, ventral. C–G, Male (MABIK CR00257794). **C** Idiosoma, dorsal; **D** Idiosoma, ventral; **E** AD, dorsal; **F** PD, dorsal; **G** GA, with branched pgs and sgs.

Idiosoma (Fig. 4C) 388 µm long (359–388 µm, mean = 372 µm, n = 5), 108 µm wide (102–137 µm, mean = 117 µm, n = 5), length-to-width ratio about 2.85–3.52; almost similar to female, except for length ratio of AD/PD and genital region. AD (Fig. 4C and E) 74 µm long (69–85 µm, mean = 76 µm, n = 5), about 0.19 times as long as idiosoma, 37 µm wide (35–43 µm, mean = 39 µm, n = 5), length-to-width ratio 1.97. PD (Fig. 4C and F) undivided, rectangular-shaped with both blunt anterior and posterior margins, almost similar to length of AD (0.96 times as long as AD), more slender 0.65 times as wide as AD; 72 µm long (68–83 µm, mean = 74 µm, n = 5), about 0.19 times as long as idiosoma, 18 µm wide (17–21 µm, mean = 19 µm, n = 5), length-to-width ratio 4.00.

GA (Fig. 4D and G) 58 µm long (52–58 µm, mean = 55 µm, n = 5), about 0.15 times as long as idiosoma, 40 µm wide (36–40 µm, mean = 38 µm, n = 5), length-to-width ratio 1.45; elliptical, with concave posterior region between GO and anal plate and gradually tapering to pointed ends at both sides posteriorly; with 7 pairs of pgs, each forming several branches towards distal part of seta. GO 19 µm long, (15–19 µm, mean = 17 µm, n = 5), 15 µm wide (12–15 µm, mean = 14 µm, n = 5), located in center of GA, about 0.33 times as long as GA; with 2 pairs of sgs, each located at anterior 45% and 74% of genital sclerites. Spermatocyte (Fig. 4D) distinct, extending beyond anterior end of PE.

#### Diagnosis

Idiosoma sub-conical, 363–372 µm long in females and 359–388 µm long in males; membranous cuticle decorated with wavy striae, except between divided AE plates; PD undivided, much more slender than AD; 5 pairs of dorsal setae (lacking ds-5); AE divided into two plates by smooth membranous cuticle; PE with 1 dorsal and 2 ventral setae; P-2 with 1 short proximal and 1 plumose distal setae, dorsally; genua I and IV with 2 and 1 ventral bipectinate setae, respectively; tibia IV with 5 setae; tarsus I with 4 ventral setae; female's GP divided into three plates; female with 1 pair of sgs on genital foramen; male's GA with 7 pairs of branched pgs and 2 pairs of sgs.

#### Etymology

The specific name is derived from the Latin ‘*angustus*’ (meaning narrow or slender), alluding to a slender posterior dorsal plate in the new species.

#### Distribution

South Korea (this study).

#### Co-occurrence

*Anomalohalacarusangustus* sp. nov. was found among particles of various sizes, ranging from fine to coarse sandy beaches, at Pohang on the east coast and Geoje and Tongyeong on the south coast. On the fine sandy beach at Pohang, *Actacarusornatus* and *Actacaruspacificus* co-occurred with the new species and on the coarse sandy beaches of Geoje and Tongyeong, *Acarochelopodia* sp., *Actacarusornatus*, *Actacaruspacificus*, and *Maracarus* sp. also were present. Harpacticoids, nematodes, polychaetes, and ostracods were observed along with halacarids.

### Anomalohalacarus biformis

Abé, 1996

63405906-683F-56D0-8EC6-004E129F74D5

*Anomalohalacarusbiformis* Abé 1996a: 8, figs. 1–4.

#### Materials

- **Type status:** Other material. **Occurrence:** individualCount: 1; sex: female; lifeStage: adult; preparations: mounted in glycerine on H-S slide; occurrenceID: 11FB49B6-4A93-5B4A-AC25-CBF05899B385; **Taxon:** scientificName: *Anomalohalacarusbiformis*; kingdom: Animalia; phylum: Arthropoda; class: Arachnida; order: Trombidiformes; family: Halacaridae; genus: Anomalohalacarus; specificEpithet: *biformis*; scientificNameAuthorship: Abé, 1996; **Location:** higherGeography: Northwest Pacific Ocean; continent: East Asia; country: Korea (the Republic of); countryCode: KR; stateProvince: Jeju-do; municipality: Seogwipo-city; locality: Jungmun-saekdal Beach, Saekdal-dong; verbatimDepth: 0.5–1 m; verbatimCoordinates: 33°14'42"N 126°24'39"E; **Identification:** identifiedBy: Shin, Chang & Lee; dateIdentified: 2024; **Event:** samplingProtocol: suction; eventDate: 22/06/2022; **Record Level:** institutionID: MABIK CR00257798; institutionCode: Marine Biodiversity Institute of Korea; basisOfRecord: PreservedSpecimen
- **Type status:** Other material. **Occurrence:** individualCount: 1; sex: female; lifeStage: adult; preparations: mounted in glycerine on H-S slide; occurrenceID: AF088E60-3754-5280-B937-8D2A8154A5FA; **Taxon:** scientificName: *Anomalohalacarusbiformis*; kingdom: Animalia; phylum: Arthropoda; class: Arachnida; order: Trombidiformes; family: Halacaridae; genus: Anomalohalacarus; specificEpithet: *biformis*; scientificNameAuthorship: Abé, 1996; **Location:** higherGeography: Northwest Pacific Ocean; continent: East Asia; country: Korea (the Republic of); countryCode: KR; stateProvince: Jeju-do; municipality: Seogwipo-city; locality: Jungmun-saekdal Beach, Saekdal-dong; verbatimDepth: 0.5–1 m; verbatimCoordinates: 33°14'42"N 126°24'39"E; **Identification:** identifiedBy: Shin, Chang & Lee; dateIdentified: 2024; **Event:** samplingProtocol: suction; eventDate: 22/06/2022; **Record Level:** institutionID: MABIK CR00257799; institutionCode: Marine Biodiversity Institute of Korea; basisOfRecord: PreservedSpecimen
- **Type status:** Other material. **Occurrence:** individualCount: 1; sex: male; lifeStage: adult; preparations: mounted in glycerine on H-S slide; occurrenceID: 0D79C7CD-B876-5B77-A698-15C33C0F830C; **Taxon:** scientificName: *Anomalohalacarusbiformis*; kingdom: Animalia; phylum: Arthropoda; class: Arachnida; order: Trombidiformes; family: Halacaridae; genus: Anomalohalacarus; specificEpithet: *biformis*; scientificNameAuthorship: Abé, 1996; **Location:** higherGeography: Northwest Pacific Ocean; continent: East Asia; country: Korea (the Republic of); countryCode: KR; stateProvince: Jeju-do; municipality: Seogwipo-city; locality: Jungmun-saekdal Beach, Saekdal-dong; verbatimDepth: 0.5–1 m; verbatimCoordinates: 33°14'42"N 126°24'39"E; **Identification:** identifiedBy: Shin, Chang & Lee; dateIdentified: 2024; **Event:** samplingProtocol: suction; eventDate: 22/06/2022; **Record Level:** institutionID: MABIK CR00257800; institutionCode: Marine Biodiversity Institute of Korea; basisOfRecord: PreservedSpecimen
- **Type status:** Other material. **Occurrence:** individualCount: 1; sex: male; lifeStage: adult; preparations: mounted in glycerine on H-S slide; occurrenceID: 2693CF12-5FB3-52F1-8AF8-582B7C2FCAAE; **Taxon:** scientificName: *Anomalohalacarusbiformis*; kingdom: Animalia; phylum: Arthropoda; class: Arachnida; order: Trombidiformes; family: Halacaridae; genus: Anomalohalacarus; specificEpithet: *biformis*; scientificNameAuthorship: Abé, 1996; **Location:** higherGeography: Northwest Pacific Ocean; continent: East Asia; country: Korea (the Republic of); countryCode: KR; stateProvince: Jeju-do; municipality: Seogwipo-city; locality: Jungmun-saekdal Beach, Saekdal-dong; verbatimDepth: 0.5–1 m; verbatimCoordinates: 33°14'42"N 126°24'39"E; **Identification:** identifiedBy: Shin, Chang & Lee; dateIdentified: 2024; **Event:** samplingProtocol: suction; eventDate: 22/06/2022; **Record Level:** institutionID: MABIK CR00257801; institutionCode: Marine Biodiversity Institute of Korea; basisOfRecord: PreservedSpecimen

#### Description

**Female**

**Figure 5.**
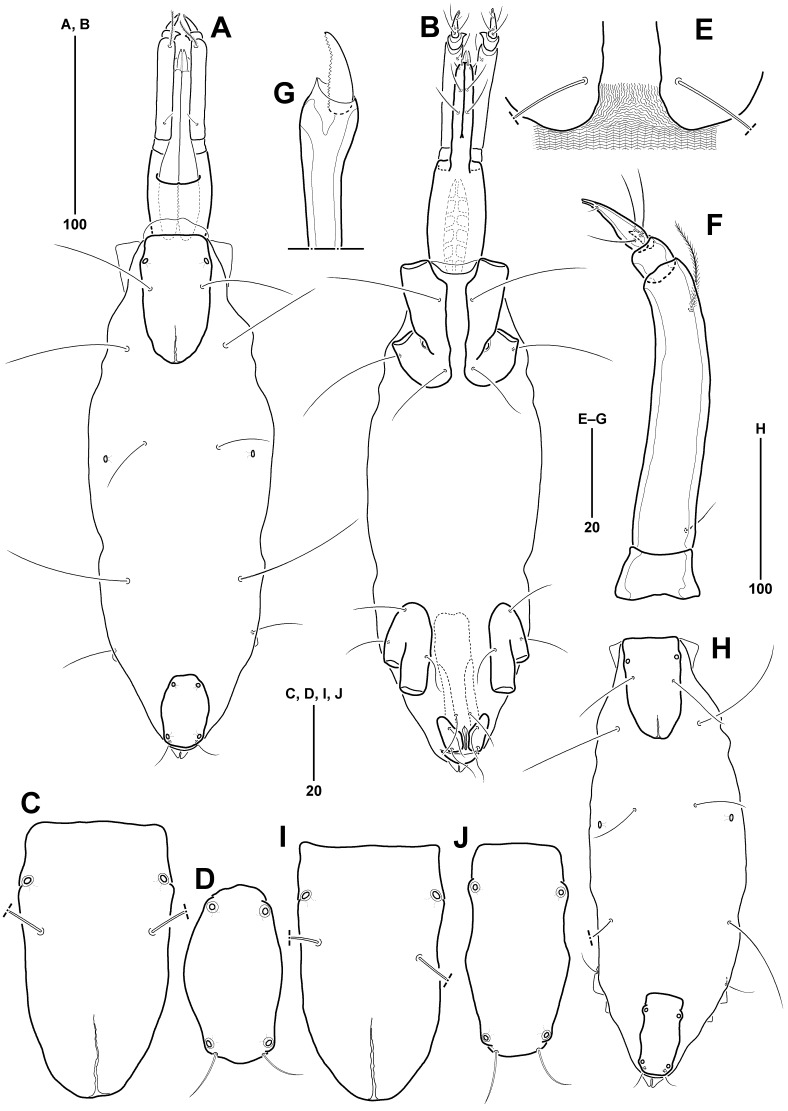
*Anomalohalcarusbiformis* Abé, 1996. A–G, Female (MABIK CR00257798). **A** Idiosoma, dorsal; **B** Idiosoma, ventral; **C** AD, dorsal; **D** PD, dorsal; **E** AE, ventral; **F** Palp, lateral; **G** Tip of chelicerae, lateral. H–J, Male (MABIK CR00257800). **H** Idiosoma, dorsal; **I** AD, dorsal; **J** PD, dorsal.

Idiosoma (Fig. 5A) elongate, 298 µm long (284–298 µm, mean = 291 µm, n = 2), 103 µm wide (98–103 µm, mean = 101 µm, n = 2), length-to-width ratio about 2.89 (2.89–2.90, mean = 2.90, n = 2). AD and PD clearly separated by membranous cuticle with transversely parallel wavy striae. OC absent. Membranous cuticle between AD and PD occupying 59% of idiosoma; decorated with three pairs of ds (ds-2 to ds-4) and 1 pair of glp-2 situated at anterior 42% of idiosoma dorsolaterally.

AD (Fig. 5A and C) 67 µm long (58–67 µm, mean = 63 µm, n = 2), about 0.22 times as long as idiosoma, 36 µm wide (32–36 µm, mean = 40 µm, n = 2), length-to-width ratio 1.86; rectangular in shape with truncated anterior margin and slightly convex posterior margin; ornamented with a longitudinally weakly split areolation in the middle to posterior third of AD, 1 pair of glp-1 at anterior 21% of AD laterally, followed by 1 pair of ds-1.

PD (Fig. 5A, D) undivided, 43 µm long (39–43 µm, mean = 41 µm, n = 2), about 0.14 times as long as idiosoma, 23 µm wide (21–23 µm, mean = 22 µm, n = 2), length-to-width ratio 1.87; smaller than AD, 0.64 times as long as AD, 0.64 times as wide as AD; convex anterior and lateral margins, and slightly blunt posterior margin; with 2 pairs of glp (glp-3 and glp-4) placed at anterior 12% and 87% of PD, respectively and 1 pair of ds-6 at posterior glp-4.

Five pairs of dorsal setae (Fig. 5A): ds-1 positioned at anterior 40% of AD laterally; 3 pairs of ds (ds-2 to ds-4) more and less evenly spaced on membranous cuticle at 20%, 38%, and 63% of idiosoma, respectively; ds-3 shorter than ds-2 and ds-4; ds-5 absent; ds-6 shortest, situated at posterior edge of PD.

All ventral plates (Fig. 5B) small and separated by membranous cuticle with striae, excluding between left and right plates of AE. AE (Fig. 5B) 69 µm long (58–69 µm, mean = 64 µm, n = 2), about 0.23 times as long as idiosoma, 32 µm wide (26–32 µm, mean = 29 µm, n = 2), length-to-width ratio 2.16; divided into left and right plates by smooth membranous cuticle (Fig. 5E), of which membranous cuticle with striae extending posterior ventral setae about 1/6 posterior part; distance between plates widest, 24 μm near insertion of leg I and narrowing gradually towards posterior part; each plate bearing 2 ventral, 1 lateral setae, and 1 epimeral pore; posterior ventral seta (41 µm long) shorter than others; epimeral pore inserted at border of epimera I and II, about anterior 66% of AE.

PE (Fig. 5B) 48 µm long (45–48 µm, mean = 47 µm, n = 2), about 0.16 times as long as idiosoma, 21 µm wide (20–21 µm, mean = 21 µm, n = 2), length-to-width ratio 2.29; situated at anterior 63–81% of idiosoma; each plate bearing 1 dorsolateral, 2 ventral setae and 1 vestigial scar of seta; ventral setae located at anterolateral corner and at half of PE, respectively.

**Figure 6.**
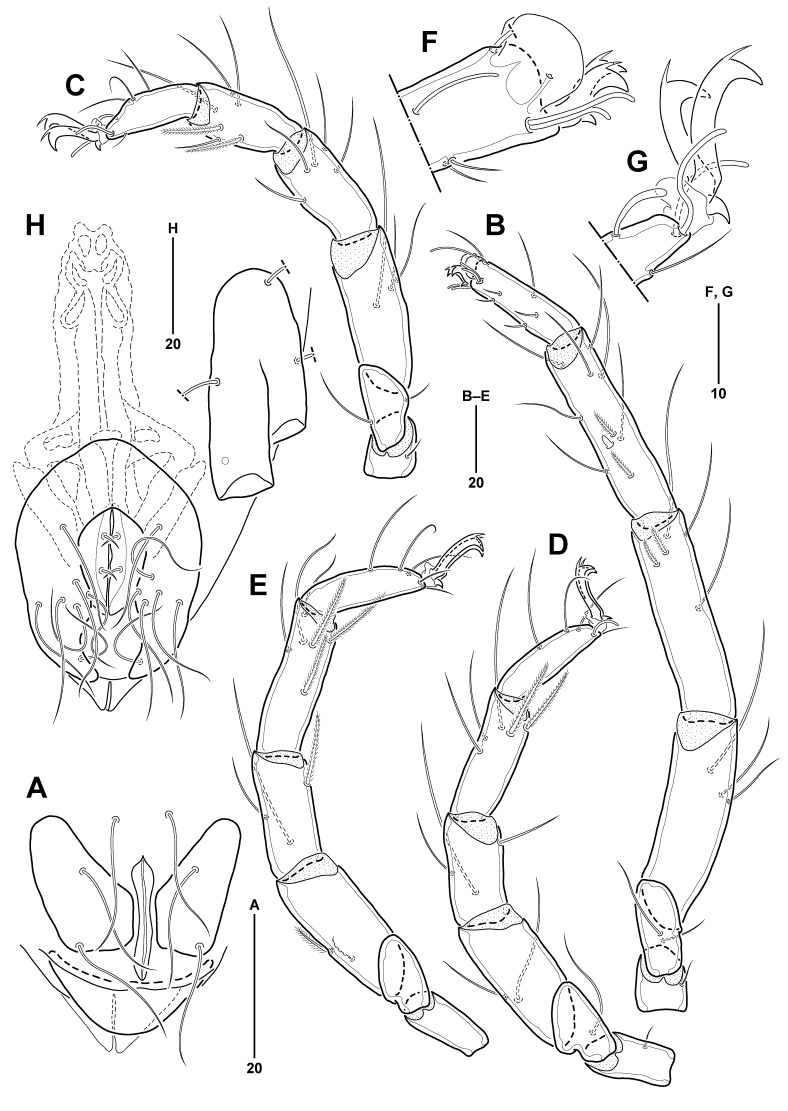
*Anomalohalcarusbiformis* Abé, 1996. A–G, Female (MABIK CR00257798). **A** GP, ventral; **B–E** Legs I–IV, ventromedial; **F** Tip of tarsus I, lateral; **G** Tip of tarsus II, lateral. **H** Male (MABIK CR00257800), left of PE, GP and spermatocyte.

GP (Fig. 6A) divided into 3 plates, consisting of 1 pair of anterior and 1 posterior genital plates; anterior genital plates sub-rectangular-shaped, located on either side of genital foramen, 18 µm long (12–18 µm, mean = 15 µm, n = 2), 11 µm wide (8–11 µm, mean = 10 µm, n = 2), length-to-width ratio of 1.64; posterior genital plate inverted triangular posterior to genital foramen, 7 µm long (n = 2), 22 µm wide (20–22 µm, mean = 21 µm, n = 2), length-to-width ratio of 0.32; 3 pairs of pgs, consisting of foremost one located on membranous cuticle anterior to genital foramen and the other 2 pairs on anterior genital plates. Genital foramen (Fig. 6A) 17 µm long (15–17 µm, mean = 16 µm, n = 2), 3 µm wide (n = 2); slender, pointed at both anterior and posterior tips, widest at anterior 15% of genital foramen; sgs absent; genital groove placed between posterior genital plate and anal foramen. Ovipositor (Fig. 5B) tube-shaped, anterior end not reaching anterior edge of PE.

Gnathosoma (Fig. 5A and B) 118 µm long (114–118 µm, mean = 116 µm, n = 2), about 0.36 times as long as idiosoma, 31 µm wide (29–31 µm, mean = 30 µm, n = 2), length-to-width ratio of 3.81; without ornamentation on surface. Rostrum (Fig. 5B) 59 µm long, slightly longer than gnathosomal base, its anterior tip not extending beyond distal end of P-2; 4 pairs of rostral setae, composing of 2 pairs of short proto- and deutorostral setae situated on distal end of rostrum and 2 pairs of long trito- and basirostral setae at anterior 29% and 59% of rostrum ventrally, respectively. Palp (Fig. 5F) 4-segmented, 11, 58, 9, and 15 µm long, respectively; P-1 without setae and spines; P-2 longest, with 1 short proximal and 1 plumose distal setae, dorsally; P-3 shortest, with 1 dorsomedial spine; P-4 conical, its terminal end bifurcated, with 3 proximal and 1 tiny distal setae. Chelicera (Fig. 5A and G) 119 µm long, consisting of basal and movable distal segments; basal segment 107 µm long, with wide proximal part and slender distal part (38%); movable segment 20 µm long, with about 25 fine denticles dorsally. Tectum (Fig. 5A) tiny, weakly convex at middle of anterior edge.

All legs (Fig. 6B–E) slender, leg I longest, 257 µm long, and legs II–IV 174 µm, 175 µm, and 186 µm long (0.67, 0.68, and 0.72 times as long as leg I), respectively. Chaetotaxy for legs I–IV as in *Anomalohalacarusangustus* sp. nov., except for tibiae 10-6-5-6(5); arrangement of legs I–IV similar to the new species. All legs with a pair of lateral claws bearing 1 accessory process dorsally and with single median claw; lateral claws of tarsus I shortest.

**Male**

Idiosoma (Fig. 5H), 294 µm long (294–311 µm, mean = 303 µm, n = 2), 102 µm wide (102–110 µm, mean = 106 µm, n = 2), length-to-width ratio about 2.83–2.88; almost similar to female, except for length ratio of AD/PD and genital region. AD (Fig. 5H and I) 62 µm long (62–76 µm, mean = 69 µm, n = 2), about 0.21 times as long as idiosoma, 35 µm wide (35–42 µm, mean = 39 µm, n = 2), length-to-width ratio 1.77. PD (Fig. 5H and J) 52 µm long (52–63 µm, mean = 58 µm, n = 2), about 0.18 times as long as idiosoma, 26 µm wide (26–32 µm, mean = 29 µm, n = 2), length-to-width ratio 2.00; undivided, rectangular with truncated anterior and posterior margins; smaller than AD, 0.84 times as long as AD and 0.74 times as wide as AD.

GA (Fig. 6H) 50 µm long (50–57 µm, mean = 54 µm, n = 2), about 0.17 times as long as idiosoma, 38 µm wide (38–43 µm, mean = 41 µm, n = 2), length-to-width ratio 1.32; elliptical, with a depressed posterior region behind GO and gradually tapering towards posterior ends at both sides; with 7 pairs of filiform pgs, foremost pgs at level of anterior sgs. GO (Fig. 6H) 20 µm long, (20–26 µm, mean = 23 µm, n = 2), about 0.40 times as long as GA, 12 µm wide (12–17 µm, mean = 15 µm, n = 2); with 2 pairs of sgs, each situated at anterior 26% and 58% of genital sclerites, respectively. Spermatocyte (Fig. 6H) extending beyond anterior end of PE.

#### Distribution

Japan (Abé 1996a), Korea (this study).

#### Co-occurrence

*Anomalohalacarusbiformis* occurred at a beach on the southern coast of Jeju Island, South Korea. Specimens were obtained by filtering sand with medium-sized grains, along with *Actacaruspacificus*, *Acarochelopodia* sp., *Acaromantis* sp., *Copidognathus* sp., *Maracarus* sp., *Rhombognathus* sp., *Simognathus* sp., and *Scaptognathus* sp. In addition to halacarids, harpacticoid copepods, nematodes, polychaetes, and ostracods were also present in the sand sediments.

## Identification Keys

### Key to species of the genus *Anomalohalacarus*, including a new species from Korea

**Table:** 

1	PD divided into two plates	2
–	PD undivided	6
2	PE with two ventral setae; P-2 with two setae	3
–	PE with three ventral setae; P-2 with one seta	*A.marcandrei*
3	Tibia IV with three bipectinate setae; tarsus I with four ventral setae	4
–	Tibia IV with two bipectinate setae; tarsus I with two setae and one spine, ventrally	5
4	AD with longitudinal areolation posteriorly; male with branched pgs	*A.angustus* sp. nov.
–	AD without areolations posteriorly; male with filiform pgs	*A.biformis*
5	Tibia I with two bipectinate setae	*A.dampierensis*
–	Tibia I without bipectinate setae	*A.macellus*
6	P-2 with one seta	7
–	P-2 with two setae	19
7	AE divided into two plates	8
–	AE undivided	*A.singularis*
8	Tibia I with one ventral small spine	9
–	Tibia I without ventral small spines	15
9	Tarsus II with one ventral seta	10
–	Tarsus II without ventral setae	*A.minutus*
10	Genu I with five setae	11
–	Genu I with four setae	*A.ruffoi*
11	Telofemur II with four setae; tibia II with five setae	12
–	Telofemur II with three setae; tibia II with six setae	*A.septentrionalis*
12	Telofemur I with four setae	13
–	Telofemur I with three setae	*A.arenarius*
13	Trochanter IV without setae	14
–	Trochanter IV with one seta	*A.poizati*
14	Rostrum extending to the distal end of P-2; tibia I with seven setae	*A.tenellus*
–	Rostrum does not extend to the distal end of P-2; tibia I with eight setae	*A.tenuis*
15	Basifemora I and II with two setae, respectively	16
–	Basifemora I and II with one seta, respectively	*A.mollis*
16	Trochanter IV without setae; telofemur II with three setae	17
–	Trochanter IV with one seta; telofemur II with four setae	*A.acnemus*
17	Trochanter III with one seta	18
–	Trochanter III without setae	*A.intermedius*
18	Genua I and II with five and four setae, respectively	*A.affinis*
–	Genua I and II with four and three setae, respectively	*A.similis*
19	Tarsus I with four ventral setae; tarsi III and IV with four dorsal setae, respectively	*A.anomalus*
–	Tarsus I with two setae and one spine, ventrally; tarsi III and IV with three dorsal setae, respectively	*A.litoralis*

## Discussion

To date, 19 species have been recognised in the genus *Anomalohalacarus* since Newell (1949) established the genus to accommodate *A.anomalus* (Trouessart, 1894) (= *Halacarusanomalus*), based on characteristics such as both PD and AE being divided into two plates longitudinally, a straight palp, P-2 with one dorsal seta, and PE with one dorsal and three ventral setae (Newell 1949). Among these diagnostic characteristics, the divided PD trait was noted initially. However, after individuals of *A.anomalus* with undivided PD were reported from France, Spain, Algeria, and Italy (Angelier 1954, Morselli 1969), the undivided PD trait was also accepted. Four of the 19 species exhibit undivided PD: *A.biformis* Abé, 1996, *A.dampierensis* Bartsch, 2003, *A.macellus* Bartsch, 1993, and *A.marcandrei* (Monniot, 1967) (Monniot 1967, Bartsch 1993, Abé 1996a, Bartsch 2003, WoRMS 2024).

The two species, discovered on intertidal sandy beaches in South Korea, *Anomalohalacarusangustus* sp. nov. and *A.biformis* Abé, 1996, display the main characteristics of the genus *Anomalohalacarus*, such as a slender idiosoma, reduced AD and PD, absent OC and corneae, the AE divided into paired left and right plates longitudinally, a slender gnathosoma, the female GP consisting of a pair of anterior and one posterior genital plates, and three pairs of pgs in the female (Bartsch 1981, Bartsch 1993, Abé 1996a). *Anomalohalacarusangustus* sp. nov. shows the greatest morphological similarity to *A.biformis* among the four species with undivided PD in sharing three and two pairs of ventral setae on AE and PE, respectively, two setae on P-2, a ventral seta on tibia I, three bipectinate setae on tibia IV, four ventral setae on tarsus I, the arrangement of pgs in the female, and two pairs of sgs in the male. However, the new species is discriminated from *A.biformis* by the following characteristics: (1) the absence of a tiny longitudinal areolation on the posterior AD [vs. the presence of the areolation in *A.biformis* (Abé 1996a, Fig. 1A)]; (2) a slender PD with a length-to-width ratio of about 3:1 in the female (about 1:1 in *A.biformis*); (3) a single pair of sgs at the genital sclerites in the female (vs. lacking sgs in *A.biformis*); and (4) all branched pgs in the male (vs. simple filiform pgs in *A.biformis*). The most notable characteristic of *A.angustus* sp. nov. is the presence of branched pgs in the male reproductive area, which has never been observed in male members of *Anomalohalacarus* and is documented for the first time in this genus. This character also appears in some species of the genera *Acaromantis* Trouessart and Neumann, 1893, *Agaue* Lohmann, 1889, *Agauopsis* Viets, 1927, *Rhombognathus* Trouessart, 1888, and *Simognathus* Trouessart, 1889 (Abé 1996b, Abé 1998, Bartsch 2006, Bartsch 2015).

Besides the unique feature of branched pgs in the new species, the remaining three species with the undivided PD differ from the new species by the presence of a sub-oval shaped PD (vs. a slender sub-rectangular shaped PD in the new species), presence of a spine and two setae on tarsus I ventrally (vs. only four ventral setae in the new species), absence of ventral setae on tarsus II (vs. one seta in the new species), and the armature of ventral spines on tibia I (two in *A.dampierensis* and *A.macellus*, absent in *A.marcandrei* vs. one in the new species). Moreover, *A.marcandrei* is easily distinguished from *A.angustus* sp. nov. by having six pairs of dorsal setae on the idiosoma (vs. five setae in the new species), two pairs of setae on AE (vs. three setae in the new species), four pairs of setae on PE (vs. three setae in the new species), one seta on P-2 (vs. two setae in the new species), and no sgs in either sex (vs. one and two sgs in the female and male, respectively, in the new species). *Anomalohalacarusdampierensis* and *A.macellus*, recorded from Australia, are discernible from *A.angustus* sp. nov. by the following differences: (1) the two species exhibit zero and two bipectinate setae on telofemur IV and tibia IV, respectively, whereas *A.angustus* sp. nov. has one and three bipectinate setae, respectively; (2) *A.dampierensis* possesses eight pgs in the male, whereas *A.angustus* sp. nov. has 14 pgs; and (3) the arrangement of the pgs in the female (anterior to GP/on GP/posterior to GP) is 1/1/1 in *A.macellus*, but 1/2/0 in the new species.

The Korean specimens of *A.biformis* coincide with the original description of Japanese specimens by Abé (1996a), except for bearing two bipectinate setae on tibia II in all Korean specimens (vs. only one bipectinate seta in the Japanese specimens). The total number of setae on tibia II ranges from five to six, which corresponds to the range of variation observed in the Japanese specimens. Abé (1996a) noted that the anterior genital plate of *A.biformis* has two shapes, sub-rectangular and sub-triangular, as indicated by the epithet. In all Korean specimens, only the sub-rectangular anterior genital plate was observed.

## Supplementary Material

XML Treatment for Anomalohalacarus angustus

XML Treatment for Anomalohalacarus biformis
